# Prognostic Value of Cadmium-Zinc-Telluride Dedicated Cardiac SPECT Dynamic Myocardial Perfusion Quantitative Imaging in Patients with Coronary Chronic Total Occlusion: A Pilot Study

**DOI:** 10.3390/jcdd13030118

**Published:** 2026-03-04

**Authors:** Linlin Li, Zekun Pang, Jianming Li, Wengui Xu

**Affiliations:** 1 Department of Molecular Imaging and Nuclear Medicine, Tianjin Medical University Cancer Institute and Hospital, National Clinical Research Center for Cancer, Huanhuxi Road, Hexi District, Tianjin 300060, China; medimaginglee@tmu.edu.cn; 2 Tianjin’s Clinical Research Center for Cancer, Tianjin 300060, China; 3 Key Laboratory of Cancer Immunology and Biotherapy, Tianjin Medical University, Ministry of Education, Tianjin 300060, China; 4 Department of Nuclear Medicine, TEDA International Cardiovascular Hospital, No. 61, 3rd Street, Binhai New Area, Tianjin 300457, China; kimi0925@126.com

**Keywords:** cadmium-zinc-telluride, myocardial blood flow quantification, chronic total occlusion, risk stratification, prognostic value

## Abstract

Background: The prevalence of chronic total occlusion (CTO) lesions is as high as 30% in patients undergoing coronary angiography (CAG). Some CTO patients do not undergo revascularization due to procedural complexity and high risks. This study aimed to investigate the value of cadmium-zinc-telluride (CZT) SPECT dynamic myocardial perfusion imaging (MPI) for risk stratification and prognosis assessment in patients with coronary CTO. Methods: This study retrospectively included 62 patients who underwent CZT SPECT dynamic MPI examination and were diagnosed with CTO by angiography. The primary endpoint was major adverse cardiovascular events (MACEs), defined as cardiovascular death, non-fatal myocardial infarction, non-fatal stroke, hospitalization for heart failure, late coronary revascularization, or hospitalization for unstable angina. Results: Over a median follow-up of 17 months (IQR 11–23), 15 MACEs occurred. The stress myocardial blood flow (sMBF) and coronary flow reserve (CFR) in the CTO territory were significantly lower in the MACEs group compared to the non-MACEs group (all *p* < 0.05). Receiver operating characteristic analysis determined the optimal cut-off values for predicting MACEs as sMBF < 0.75 (sensitivity 78.7%, specificity 73.3%, AUC = 0.74, *p* < 0.05) and CFR < 1.39 (sensitivity 70.2%, specificity 80.0%, AUC = 0.75, *p* < 0.01). Kaplan–Meier survival analysis showed that patients with impaired sMBF (*p* < 0.001) or impaired CFR (*p* < 0.01), defined by these cut-off values, had significantly worse clinical outcomes. Conclusions: The results of this study indicate that sMBF and CFR obtained from CZT SPECT dynamic MPI provide valuable prognostic prediction for patients with coronary CTO lesions, offering critical evidence for identifying high-risk patients requiring active intervention.

## 1. Introduction

Coronary chronic total occlusion (CTO) is defined as an occlusion of an epicardial coronary artery with TIMI flow grade 0, estimated to be present for at least 3 months. The benefit of revascularization for CTO patients remains debated, although most studies suggest that revascularization can improve symptoms and prognosis, contingent upon the presence of viable myocardium, which relies on intact coronary microcirculation structure and function [1,2,3]. The distal microcirculation in CTO adapts to low perfusion by maximally dilating to maintain blood supply to viable myocardium at rest, but this leads to a reduction in coronary flow reserve (CFR) [4,5]. Notably, CTO lesions involve not only epicardial vessel occlusion but are often accompanied by microvascular dysfunction distal to the occlusion [6,7]. CFR comprehensively reflects the integrated flow regulation capacity of both the epicardial coronary arteries and the microcirculation. Therefore, early and accurate detection of CFR is crucial for risk stratification and prognosis assessment in CTO patients.

Historically, assessment of coronary blood flow and CFR was only feasible through invasive means. Due to limitations such as invasiveness, complexity, cost, and operator dependency, these methods have not been widely adopted in clinical practice [8]. As early as 2003, the American College of Cardiology (ACC), American Heart Association (AHA), and American Society of Nuclear Cardiology (ASNC) incorporated radionuclide myocardial perfusion imaging (MPI) into guidelines for coronary artery disease (CAD) management [9,10]. Recently, emerging CZT dedicated cardiac SPECT dynamic MPI, utilizing dynamic acquisition during rest and stress, not only provides conventional MPI parameters but also offers key quantitative parameters such as rest/stress myocardial blood flow (MBF) and CFR. This technique achieves performance comparable to positron emission tomography (PET) for absolute myocardial flow quantification and shows unique advantages in accurate CFR measurement [11,12,13]. It is non-invasive, clinically convenient, benefits from readily available radiopharmaceuticals, has relatively lower equipment and examination costs, and involves lower radiation doses, holding broad prospects for clinical application and dissemination.

However, its prognostic value in the specific population of CTO patients remains unclear. Searches of the literature indicate that no prior studies have systematically investigated the diagnostic value of absolute flow quantification parameters measured by CZT SPECT for prognosis assessment in CTO patients. Therefore, this study, by collecting and analyzing absolute myocardial flow parameters obtained from CZT SPECT and follow-up data from CTO patients, aims to evaluate the clinical application value of this technique for prognosis assessment in this patient group, providing non-invasive imaging guidance and objective evidence for individualized treatment and prognosis judgment.

## 2. Method

### 2.1. Patients

**Figure 1:**
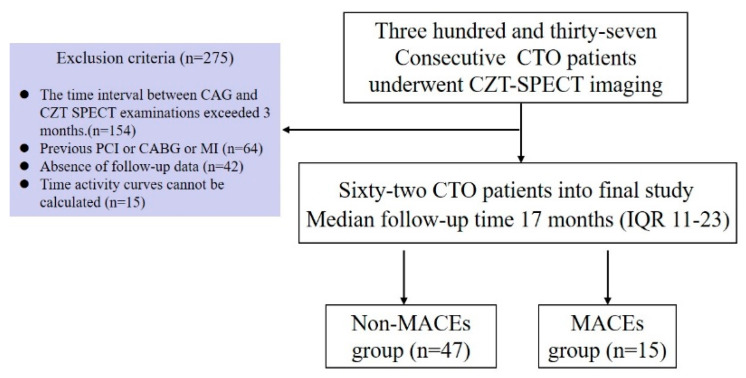
Diagram of study patient flowchart.

This retrospective study consecutively screened and analyzed clinical and imaging data from 337 patients with CTO who underwent CZT dedicated cardiac SPECT dynamic myocardial perfusion quantitative imaging between January 2022 and December 2024. Included patients had CAG confirming at least one major epicardial vessel or its major branch with TIMI flow grade 0, judged intraoperatively by an interventional cardiologist as a CTO lesion, either not attempted or failed to be opened. Exclusion criteria were: (1) time interval between CZT-SPECT and CAG exceeding 3 months (*n* = 154); (2) history of old myocardial infarction (OMI) or prior coronary artery bypass grafting (CABG) (*n* = 64); (3) incomplete, lost, or unavailable follow-up data (*n* = 42); (4) Poor quality control of CZT-SPECT flow quantification data (*n* = 15). Ultimately, 62 patients were enrolled (Figure 1). Clinical history was collected. Hypertension was defined as blood pressure ≥ 140/90 mmHg or current use of antihypertensive medication. Hypercholesterolemia was defined as total cholesterol > 6.2 mmol/L or receiving lipid-lowering therapy. Diabetes mellitus was defined as patients who were on oral hypoglycemic agents or insulin. Family history of CAD was defined as CAD in a first-degree male relative <55 years old or a female relative <65 years old. The study complied with the Declaration of Helsinki and was approved by the local ethics committee (No. 2024-1230-1). Informed consent was obtained from all patients.

### 2.2. Coronary Angiography

CAG was performed using standard techniques [14]. CAG images were independently assessed by two experienced cardiologists, with disagreements resolved by consensus. A CTO lesion was defined as a total occlusion on CAG, estimated duration ≥ 3 months, with TIMI flow grade 0 in one major coronary artery or its major branch. A diseased coronary artery was defined as an epicardial coronary artery with stenosis ≥ 50%, including native vessel or in-stent restenosis.

### 2.3. SPECT Imaging Protocol

Imaging was performed using a CZT dedicated cardiac SPECT camera (NM 530c, GE Healthcare, Haifa, Israel). The radiopharmaceutical was 99mTc-sestamibi (MIBI). Patients were instructed to discontinue relevant cardiovascular medications (nitrates for at least 6 h, calcium channel blockers and beta-blockers for at least 24 h), and to avoid caffeine-containing beverages, tea, food, and medications for at least 24 h, and methylxanthines for at least 36 h prior to the exam. All acquisitions used a “continuous rapid imaging” (CRI) protocol, detailed in our previously published literature [15].

**Figure 2:**
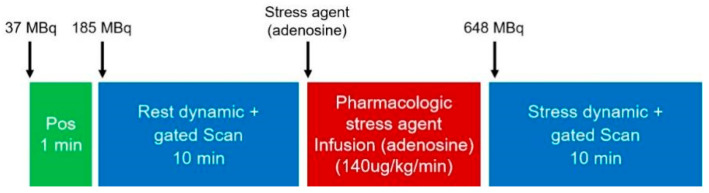
CZT-SPECT CFR continuous rapid imaging protocol.

Rest imaging was performed first. An initial bolus of approximately 37 MBq (1 mCi) of 99mTc-sestamibi was injected to aid patient positioning. Then, a low-dose of 185–259 MBq (5–7 mCi) of 99mTc-sestamibi was injected, immediately followed by a 10 min dynamic list-mode acquisition. During dynamic acquisition, ECG R-wave trigger information was simultaneously collected for synchronized dynamic/ECG-gated scanning. Stress imaging commenced immediately after rest acquisition. All patients underwent pharmacological stress with adenosine infusion (140 μg/kg/min). At the 3rd minute of adenosine infusion, a stress dose of approximately 3.5 times the rest dose (647.5–906.5 MBq [17.5–24.5 mCi]) of 99mTc-sestamibi was injected. Adenosine infusion continued for a total of 6 min. Immediately after the stress tracer injection, a 10 min synchronized dynamic/ECG-gated list-mode acquisition was performed, identical to the rest protocol. The dynamic imaging protocol is illustrated in Figure 2. Acquisition parameters were: 8 frames per cardiac cycle, heart rate acceptance window ±15%, energy peak 140 keV, and window ±10%. All patients underwent CT attenuation correction prior to SPECT imaging using a Discovery Elite PET/CT scanner (Discovery NM690, GE Healthcare, Waukesha, WI, USA) with parameters: 120 kV, 20 mA, scan range from lung apices to mid-liver.

### 2.4. SPECT Image Analysis

Image processing and interpretation were performed by two experienced nuclear medicine physicians, with disagreements resolved by consensus. MPI tomographic images and global semi-quantitative parameters, including summed stress score (SSS), summed rest score (SRS), and summed difference score (SDS), were obtained using QGS+QPS software (Version 2017, Cedars-Sinai Medical Center, Los Angeles, CA, USA) on a GE Xeleris 4DR workstation. All dynamic list-mode data were transferred to a MyoFlowQ workstation (Bailingyun Biomedical Technology Co., Ltd., Beijing, China). Dynamic data were first rebinned into a series of dynamic frames: 10 s/frame × 10 frames + 20 s/frame × 5 frames + 60 s/frame × 2 frames + 280 s/frame × 1 frame. CT attenuation correction data were applied for accurate image fusion, reorientation, attenuation, and scatter correction. Regions of interest (ROIs) for the left ventricular (LV) blood pool input function and myocardial base position were defined to generate dynamic curves and fitted curves for the LV blood pool and myocardium. After correction for residual rest activity during stress imaging, the software’s built-in model [16] was used to automatically calculate global and regional stress MBF (sMBF), rest MBF (rMBF), and their ratio, CFR (sMBF/rMBF). Based on the CAG-confirmed CTO vessel, the corresponding MBF and CFR parameters were termed CTO-rMBF, CTO-sMBF, and CTO-CFR.

### 2.5. Patients’ Follow-Up

Follow-up data were collected via telephone interviews and review of clinical records to document outcome information. The median follow-up time after CZT SPECT imaging was 17 months (IQR 11–23 months). All patients were treated with conservative drug therapy during the follow-up period. The primary endpoint, major adverse cardiac events (MACEs), was defined as the occurrence of at least one of the following: cardiovascular death, non-fatal myocardial infarction, non-fatal stroke, hospitalization for heart failure, late coronary revascularization, or hospitalization for unstable angina.

### 2.6. Statistical Analysis

Based on follow-up outcomes, patients were divided into MACE and non-MACE groups. The Kolmogorov–Smirnov test was used to assess normality. Normally distributed continuous data are presented as mean ± standard deviation (SD), non-normally distributed data as median ± IQR, and categorical data as frequencies and percentages. Categorical variables were compared using the chi-square test. Means of continuous variables between groups were compared using an independent samples *t*-test. Cumulative incidence of MACEs was assessed using the Kaplan–Meier method and compared with the log-rank test. All variables were first evaluated by univariate Cox proportional hazards regression analysis. We included variables with clinical relevance or univariate correlation with the outcome in the multivariate Cox proportional hazards regression model and constructed two models: model 1 adjusted for clinical risk factors; model 2 adjusted for traditional semi-quantitative MPI parameters. Given the limited number of events in this study (*n* = 15), to strictly adhere to statistical principles and avoid model overfitting, the number of additional predictive variables included in each model was strictly limited. Results are presented as hazard ratio (HR) and 95% confidence interval (CI). Receiver operating characteristic (ROC) curve analysis and the Youden index were used to determine the optimal cut-off values of sMBF and CFR for predicting MACEs. All data were processed using SPSS Statistics version 27.0 (IBM, Chicago, IL, USA). A *p*-value < 0.05 was considered statistically significant.

## 3. Results

### 3.1. Baseline Clinical Characteristics

Sixty-two patients with CTO met the study criteria (mean age 60.56 ± 11.80 years, 82.3% male). CAG revealed a total of 66 CTO lesions. The vast majority of patients (58/62, 93.5%) had single-vessel CTO, while 4 patients had 2 CTO lesions. CTO locations were: 13 in the LAD, 20 in the LCX, and 29 in the RCA. Based on follow-up outcomes, patients were divided into two groups: MACEs and non-MACEs. There were no significant differences in baseline clinical variables and cardiovascular risk factors between the two groups (*p* > 0.05), as shown in Table 1.

### 3.2. SPECT MPI and MBF Quantification

**Figure 3:**
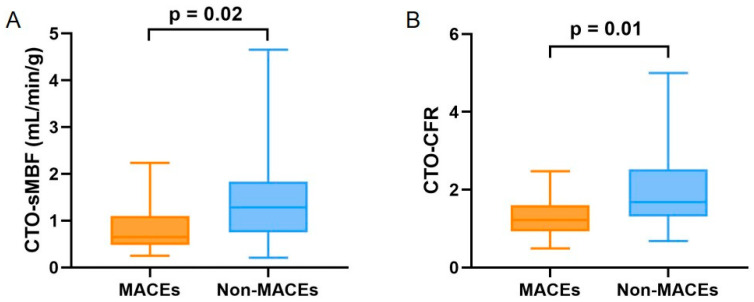
Comparison of (**A**) CTO-sMBF and (**B**) CTO-CFR between MACEs and non-MACEs groups.

The MPI and CFR results for all patients are shown in Table 2. The MACEs group had significantly lower rest LVEF (40.93 ± 17.53 vs. 51.06 ± 14.18, *p* = 0.027), rest peak ejection rate (rPER) (−1.84 ± 0.88 vs. −2.43 ± 0.86, *p* = 0.026), CTO-sMBF (0.83 ± 0.52 vs. 1.46 ± 0.93, *p* = 0.015), global LV-CFR (1.49 ± 0.68 vs. 2.06 ± 0.93, *p* = 0.031), and CTO-CFR (1.25 ± 0.49 vs. 1.96 ± 0.95, *p* = 0.007) compared to the non-MACEs group (Figure 3). Conversely, stress end-diastolic volume (sEDV) (146.27 ± 91.27 vs. 108.34 ± 50.36, *p* = 0.045), rest EDV (rEDV) (138.27 ± 76.38 vs. 103.40 ± 50.98, *p* = 0.047), stress end-systolic volume (sESV) (97.53 ± 86.71 vs. 59.43 ± 48.64, *p* = 0.035), and rest ESV (rESV) (92.80 ± 74.12 vs. 55.91 ± 47.56, *p* = 0.027) were significantly higher in the MACEs group. No significant differences were found between the groups for other parameters (*p* > 0.05). Notably, there were no significant differences in global LV-rMBF and CTO-rMBF between the two groups (*p* > 0.05).

### 3.3. Clinical Outcomes

Over a median follow-up of 17 months (IQR: 11–23), 15 patients (24.2%) experienced MACEs. These included: hospitalization for unstable angina (*n* = 6, 9.7%), hospitalization for heart failure (*n* = 5, 8.1%), hospitalization for non-fatal myocardial infarction (*n* = 3, 4.8%), and hospitalization for late revascularization (*n* = 1, 1.6%).

### 3.4. Prognostic Value of MBF Quantification

**Figure 4:**
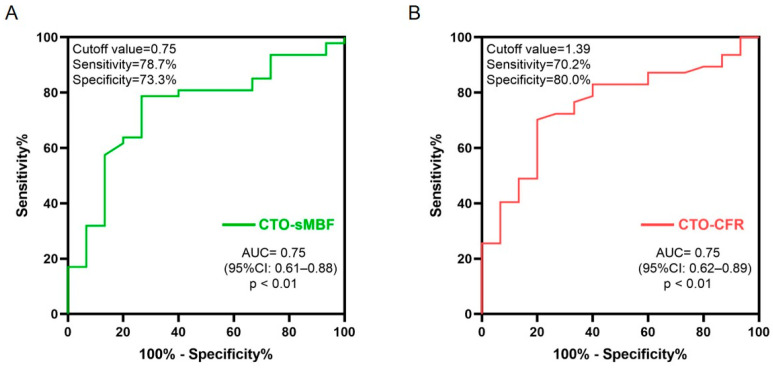
ROC curves of (**A**) sMBF and (**B**) CFR for predicting MACEs in CTO patients.

**Figure 5:**
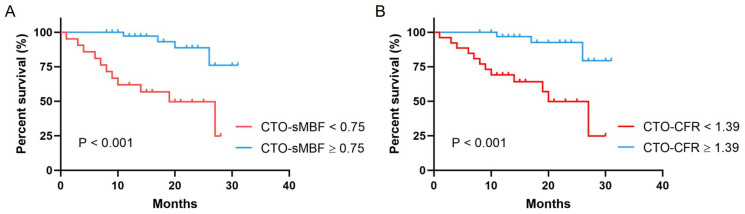
Kaplan–Meier survival curves for MACE-free survival based on (**A**) CTO-sMBF and (**B**) CTO-CFR cut-off values.

**Figure 6:**
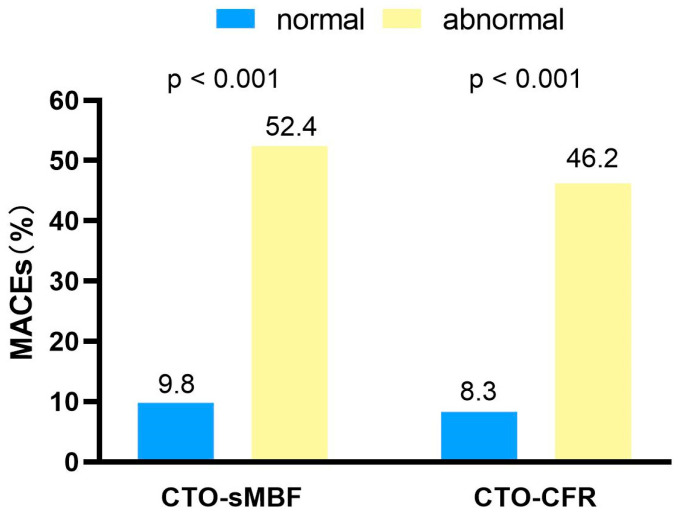
Comparison of the incidence of MACEs between the normal and abnormal groups in the CTO-sMBF and CTO-CFR.

As shown in Figure 4, ROC curve analysis indicated that the optimal cut-off values for predicting MACEs were CTO-sMBF < 0.75 (sensitivity 78.7%, specificity 73.3%, AUC = 0.74) and CTO-CFR < 1.39 (sensitivity 70.2%, specificity 80.0%, AUC = 0.75). Kaplan–Meier analysis (Figure 5) showed that patients with impaired CTO-sMBF (log-rank χ^2^ = 14.93, *p* < 0.001) and impaired CTO-CFR (log-rank χ^2^ = 13.94, *p* < 0.001) had significantly worse prognosis. Further subgroup analysis revealed that the incidence of MACEs was significantly higher in patients with abnormal CTO-sMBF and CTO-CFR compared to those with normal values: 52.4% vs. 9.8% (*p* < 0.001) and 46.2% vs. 8.3% (*p* < 0.001), respectively (Figure 6).

### 3.5. Predictive Factors of Outcomes

**Figure 7:**
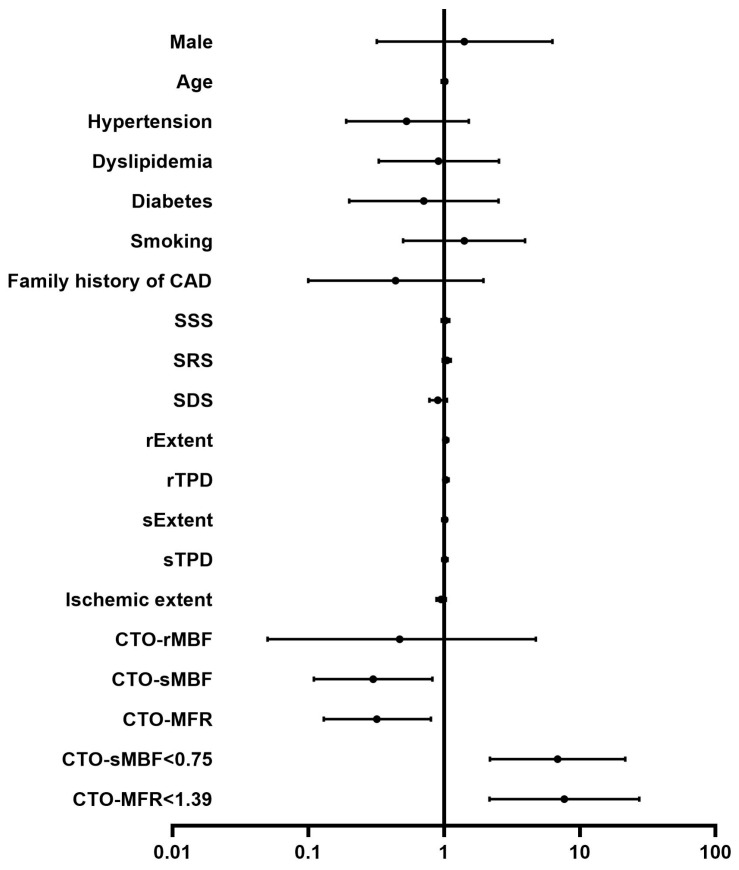
Forest plots from univariate Cox regression analysis for predictors of MACEs. The points represent the hazard ratio, and the line segments represent the 95% confidence intervals.

Univariate Cox regression analysis showed that both CTO-sMBF (HR: 0.30, 95% CI: 0.11–0.82, *p* = 0.02) and CTO-CFR (HR: 0.32, 95% CI: 0.13–0.80, *p* = 0.014) were significant predictors of MACEs (Figure 7). Multivariate Cox regression analysis results are shown in Table 3. After adjusting for clinical risk factors, both CTO-sMBF (HR: 0.26, 95% CI: 0.10–0.68, *p* = 0.006) and CTO-CFR (HR: 0.28, 95% CI: 0.11–0.67, *p* = 0.005) remained significant predictors of MACEs. After further adjusting for semi-quantitative MPI parameters (SSS, SRS, SDS), CTO-sMBF (HR: 0.21, 95% CI: 0.06–0.82, *p* = 0.025) and CTO-CFR (HR: 0.20, 95% CI: 0.05–0.72, *p* = 0.014) still remained significant independent predictors of MACEs.

## 4. Discussion

This is the first study to utilize absolute myocardial flow quantification parameters (sMBF and CFR) measured by CZT dedicated cardiac SPECT to explore their prognostic value in CTO patients. Our results demonstrate that CTO-sMBF and CTO-CFR are independent predictors of MACEs in CTO patients, with predictive capability superior to traditional semi-quantitative MPI parameters.

Recent studies suggest that although CTO patients often have well-developed collateral circulation, they are not immune to angina or even heart failure. This may be because collaterals can maintain resting perfusion but are insufficient to meet the increased myocardial blood demand during stress [17,18,19]. Further research indicates that CFR in some CTO patients is close to or even below 1, suggesting the distal vascular bed is already maximally dilated at rest and cannot further dilate to increase flow under stress. Due to reduced CFR, approximately one-third of CTO patients may exhibit the coronary “steal” phenomenon during adenosine stress [20,21]. The pathophysiological reasons for reduced CFR may involve myogenic responses, blunted NO-dependent vasodilation, and exhaustion of metabolic regulatory reserves [22,23]. By comparing myocardial blood flow between stress and rest states, CFR can effectively identify inadequate flow reserve in the collateral-dependent territory, pinpointing myocardial areas that, despite having adequate resting flow, are at risk of “hemodynamic failure.”

Currently, positron emission tomography myocardial perfusion imaging (PET MPI) is considered the non-invasive gold standard for MBF quantification. However, its clinical application is limited by scanner availability and tracer accessibility. Traditional semi-quantitative MPI provides information on myocardial perfusion and LV function but has limitations in precisely quantifying MBF due to lower spatial and temporal resolution. Compared to conventional sodium iodide (NaI) SPECT, CZT dedicated cardiac SPECT offers higher detection efficiency and spatial resolution, enabling absolute MBF quantification comparable to PET/CT, with studies showing good agreement between the two modalities [24]. Leveraging these technical advantages, CZT SPECT provides a more clinically feasible alternative for accurate CFR assessment. This study was designed based on these characteristics.

Our study found that sMBF and CFR in the CTO territory were significantly lower in the MACEs group, indicating more severe impairment in regional perfusion and flow reserve. The MACEs group also had significantly lower rest LVEF, reflecting worse LV systolic function, and larger LV volumes (sEDV, rEDV, sESV, rESV), suggesting more pronounced LV dilation and remodeling. Notably, subgroup analysis showed that the incidence of MACEs was over five times higher in patients with abnormal sMBF or CFR compared to those with normal values, with over 40% of patients in the abnormal groups experiencing MACEs. These results underscore the significant prognostic value of CTO-territory sMBF and CFR quantified by CZT SPECT. Furthermore, we found that when sMBF and CFR fell below specific cut-off values, the cumulative risk of MACEs increased steadily, providing a pathophysiological basis for risk stratification models based on these quantitative flow parameters.

The optimal cut-off values for predicting MACEs in our study were CTO-sMBF < 0.75 mL/min/g and CTO-CFR < 1.39. These thresholds are generally lower than those commonly reported in PET studies for general coronary artery disease populations, where a CFR < 2.0 is often considered indicative of impaired flow reserve and associated with worse prognosis. This discrepancy is not unexpected. CTO territories represent a unique physiological state with chronically reduced baseline flow and maximally dilated microvasculature. Therefore, the absolute values of sMBF and CFR are inherently lower. Our cut-offs should be interpreted as specific to the context of CTO pathophysiology. The key finding is that within this population, values falling below these specific thresholds identify a subgroup at significantly higher risk. This emphasizes the importance of using reference values specific to certain technologies and populations for accurate risk stratification.

Furthermore, it is worth noting that although both sMBF and CFR demonstrated powerful independent prognostic value, it is crucial to understand that they reflect distinct pathophysiological emphases. sMBF primarily represents the absolute level of blood flow achievable by the myocardial microvascular bed under pharmacologic stress. It directly reflects the maximum blood supply capacity of the entire coronary system, encompassing both the epicardial arteries and the microcirculation. A reduction in sMBF directly indicates the presence of a flow-limiting lesion, leading to myocardial ischemia. In contrast, CFR reflects the ability of the coronary system to increase blood flow from a resting state to a state of maximal hyperemia. In patients with CTO, due to the presence of collateral circulation, the rMBF might be maintained at a relatively normal level through collateral compensation. However, the dilation reserve of collateral vessels is limited, preventing a significant increase in flow during stress, which manifests as a markedly reduced CFR. Consequently, CFR is more sensitive in revealing a “decompensated” state where the flow reserve is exhausted even if resting flow is still adequate.

In clinical practice, the decision regarding whether to pursue revascularization for CTO remains controversial [25,26,27]. Although revascularization via CABG or PCI can alleviate symptoms and improve quality of life in some CTO patients, its benefit in improving long-term survival or reducing MACEs compared to optimal medical therapy alone is not clearly established. Our findings suggest that CFR can provide crucial guidance for revascularization decisions. If CFR in the CTO territory is significantly reduced, indicating substantial myocardial ischemia, these patients may derive significant symptomatic and prognostic benefit from successful revascularization. Conversely, if CFR is normal or near-normal, suggesting adequate collateral compensation and minimal ischemia, the potential benefit of high-risk revascularization may be limited, and a conservative strategy based on medical therapy may be more appropriate. It is also important to note that this study defined MACEs as a composite endpoint, which includes endpoints of varying clinical severity, such as hard endpoints (e.g., non-fatal myocardial infarction) and soft endpoints (e.g., hospitalization for unstable angina or heart failure). Although composite endpoints are commonly used to increase statistical power, we observed that the majority of events in this cohort were soft endpoints. This may reflect that, during the relatively short follow-up period, CTO lesions were more likely to lead to exacerbation of ischemic symptoms or decompensation of heart failure rather than extreme events. This suggests that the sMBF and CFR cut-off values identified in this study may hold significant value in predicting such “moderate-risk” events that require medical intervention. Future studies with larger sample sizes and longer follow-up durations are expected to further differentiate the specific predictive capabilities of these hemodynamic parameters for hard versus soft endpoints.

### Study Limitations

This study has several limitations. First, the sample size of this study is relatively small and the number of events is limited, which implies that there is a potential risk of overfitting. This may affect the accuracy of the multivariate analysis results and may limit the extrapolation of the optimal critical value to a broader population of CTO patients. Second, this study excluded patients with a history of OMI or CABG. While this design helped to obtain a “pure” CTO cohort by reducing the confounding effects of prior MI or complex revascularization history on the assessment of myocardial viability and blood flow, it introduced selection bias, thereby limiting the generalizability of the study conclusions to a certain extent. Third, we did not quantitatively assess the extent and functionality of collateral circulation. The quality of collaterals is a key factor influencing resting MBF and the capacity to increase flow during stress, thereby directly impacting CFR. The lack of collateral assessment means we cannot fully disentangle the contribution of epicardial occlusion from microvascular dysfunction mediated by poor collateral supply. Finally, the findings require external validation through larger-scale, multicenter prospective studies to further confirm the optimal cut-off values and prognostic power of CZT SPECT-derived CFR for predicting MACEs in CTO patients.

## 5. Conclusions

Our results preliminarily validate the clinical utility of absolute myocardial flow quantification parameters obtained from CZT cardiac SPECT for prognosis assessment in CTO patients. CTO-territory sMBF and CFR were identified as independent prognostic predictors for MACEs, with predictive performance superior to traditional semi-quantitative MPI parameters. This represents a shift from purely anatomical assessment towards an integrated “anatomy-function-physiology” evaluation, which is expected to become a widely available and powerful tool. By providing quantitative blood flow information similar to PET, it can guide risk stratification and management decisions for CTO patients, thereby bridging the gap in accessibility of the “gold standard” technology and allowing more patients to benefit from precise assessment.

## Figures and Tables

**Table 1:** Baseline characteristics of 62 unrevascularized CTO patients.

	Total (*n* = 62)	Non-MACEs (*n* = 47)	MACEs (*n* = 15)	*p* Value
Age (years)	60.56 ± 11.80	60.43 ± 12.56	61.00 ± 9.39	0.87
Male gender, *n* (%)	51 (82.3%)	38 (80.9%)	13 (86.7%)	0.61
Hypertension, *n* (%)	44 (71.0%)	35 (74.5%)	9 (60.0%)	0.28
Dyslipidemia, *n* (%)	30 (48.4%)	23 (48.9%)	7 (46.7%)	0.88
Diabetes, *n* (%)	19 (30.6%)	16 (34.0%)	3 (20.0%)	0.30
Smoking, *n* (%)	26 (41.9%)	18 (38.2%)	8 (53.3%)	0.30
Family history of CAD	15 (24.2%)	13 (27.7%)	2 (13.3%)	0.26

**Table 2:** CZT-SPECT MPI and MBF quantification results of 62 unrevascularized CTO patients.

	Total (*n* = 62)	Non-MACEs (*n* = 47)	MACEs (*n* = 15)	*p* Value
Stress				
EDV (mL)	117.52 ± 63.98	108.34 ± 50.36	146.27 ± 91.27	0.045
ESV (mL)	68.65 ± 61.49	59.43 ± 48.64	97.53 ± 86.71	0.035
LVEF (%)	48.03 ± 15.52	49.96 ± 14.29	42.00 ± 18.09	0.084
SSS (IQR)	10 (9)	10 (8)	10 (15)	0.792
EXT (%)	18 (21)	18 (19)	16 (31)	0.895
TPD (%)	14 (16)	14 (15)	13 (22)	0.818
PER (−EDV/s)	−2.52 ± 1.01	−2.63 ± 0.99	−2.15 ± 1.05	0.112
PFR (EDV/s)	1.93 ± 0.88	2.00 ± 0.79	1.74 ± 1.15	0.332
LV-MBF (mL/min/g)	1.48 ± 0.89	1.60 ± 0.93	1.11 ± 0.68	0.067
CTO-MBF (mL/min/g)	1.31 ± 0.89	1.46 ± 0.93	0.83 ± 0.52	0.015
Rest				
EDV (mL)	111.84 ± 59.38	103.40 ± 50.98	138.27 ± 76.38	0.047
ESV (mL)	64.84 ± 56.75	55.91 ± 47.56	92.80 ± 74.12	0.027
LVEF (%)	48.61 ± 15.53	51.06 ± 14.18	40.93 ± 17.53	0.027
SRS (IQR)	2 (8)	2 (6)	5 (16)	0.281
EXT (%)	3 (15)	2 (8)	5 (19)	0.171
TPD (%)	3 (12)	3 (9)	5 (17)	0.246
PER (−EDV/s)	−2.29 ± 0.89	−2.43 ± 0.86	−1.84 ± 0.88	0.026
PFR (EDV/s)	1.76 ± 0.77	1.83 ± 0.72	1.54 ± 0.89	0.192
LV-MBF (mL/min/g)	0.74 ± 0.18	0.74 ± 0.19	0.71 ± 0.16	0.501
CTO-MBF (mL/min/g)	0.72 ± 0.25	0.72 ± 0.25	0.69 ± 0.28	0.660
SDS (IQR)	5 (6)	6 (6)	4 (3)	0.190
Ischemic extent	10 (12)	12 (13)	8 (7)	0.141
LV-CFR	1.92 ± 0.90	2.06 ± 0.93	1.49 ± 0.68	0.031
CTO-CFR	1.79 ± 0.91	1.96 ± 0.95	1.25 ± 0.49	0.007

**Table 3:** Multivariable predictors of MACEs.

Variables	Adjust for CRF HR (95% CI)	*p* Value	Adjust for MPI HR (95% CI)	*p* Value
CTO-sMBF	0.26 (0.10–0.68)	0.006	0.21 (0.06–0.82)	0.025
CTO-CFR	0.28 (0.11–0.67)	0.005	0.20 (0.05–0.72)	0.014

CRF (clinical risk factor), including age, gender, hypertension, dyslipidemia, diabetes, smoking and family history of CAD; MPI including rExtent, rTPD, sExtent, sTPD, SSS, SRS, SDS and ischemic extent.

## Data Availability

The data presented in this study are available from the corresponding author upon reasonable request due to applicable privacy and institutional confidentiality restrictions.

## Abbreviations

The following abbreviations are used in this manuscript:

CTO	chronic total occlusion
CZT	cadmium-zinc-telluride
MACEs	major adverse cardiovascular events
MPI	myocardial perfusion imaging
CAD	coronary artery disease
CFR	coronary flow reserve
MBF	myocardial blood flow
OMI	old myocardial infarction
SSS	summed stress score
SRS	summed rest score
SDS	summed rest score
CABG	coronary artery bypass grafting
